# Association between Five Established DASH Diet Indices and Risk of Type 2 Diabetes Mellitus: A Population-Based Prospective Cohort Study

**DOI:** 10.34172/aim.34602

**Published:** 2025-11-01

**Authors:** Hanieh Malmir, Somayeh Hosseinpour-Niazi, Parvin Mirmiran, Fereidoun Azizi

**Affiliations:** ^1^Nutrition and Endocrine Research Center, Research Institute for Endocrine Disorders, Research Institute for Endocrine Sciences, Shahid Beheshti University of Medical Sciences, Tehran, Iran; ^2^Department of Clinical Nutrition and Dietetics, Faculty of Nutrition Sciences and Food Technology, National Nutrition and Food Technology Research Institute, Shahid Beheshti University of Medical Sciences, Tehran, Iran; ^3^Endocrine Research Center, Research Institute for Endocrine Disorders, Research Institute for Endocrine Sciences, Shahid Beheshti University of Medical Sciences, Tehran, Iran

**Keywords:** Dietary approach to stop hypertension, Dixon index, Günther, Index, Type 2 diabetes

## Abstract

**Background:**

Type 2 diabetes mellitus (T2DM) represents a rising international health crisis, closely linked to changing eating habits and lifestyle choices. The Dietary Approaches to Stop Hypertension (DASH) diet has gained interest due to its potential to avert and control insulin resistance and T2DM. This research aims to investigate the association between five DASH diet indices and the risk of T2DM within the Tehran Lipid and Glucose Study (TLGS).

**Methods:**

Individuals aged 21 years and older, who were free from T2DM at baseline, were included. Dietary habits were evaluated using a validated food frequency questionnaire; adherence to the DASH diet was assessed through five indices: Folsom, Dixon, Mellen, Fung, and Günther. To assess the association between the DASH diet and T2DM risk, Cox proportional hazards models, adjusted for demographic & lifestyle factors, were employed.

**Results:**

Of the 2,188 participants, 989 (45.2%) were men. The mean (SD) age and BMI of the population were 40.6 (13.3) years and 27.0 (4.7) kg/m^2^, respectively. During a median follow-up of 8.9 years, 234 new cases of T2DM were recorded. During a median follow-up period of 8.9 years, 234 new T2DM cases surfaced. Initially, no association was found in crude models; however, after adjustments for confounders, higher adherence to the Dixon index (HR=0.71; 95% CI: 0.52–0.98) and Günther index (HR=0.69; 95% CI: 0.50–0.96) were associated with a lower risk of T2DM. This association remained significant for the Günther index among those who were physically active (HR=0.70; 95% CI: 0.51–0.97).

**Conclusion:**

High adherence to DASH diets, evaluated using the Dixon and Günther DASH indices, is associated with a lower risk of T2DM, particularly among individuals with low activity levels; therefore, the inclusion of the DASH diet, especially for those with low physical activity, is recommended as a component of public health strategies for T2DM prevention in Tehrani adults.

## Introduction

 Type 2 diabetes mellitus (T2DM) is an increasingly significant health concern affecting millions of individuals worldwide.^1^ In 2021, nearly 537 million individuals aged 18 years and older were living with T2DM, according to reports from the International Diabetes Federation (IDF). If current trends continue, this number is projected to increase to 783 million by 2045.^2^ The Middle East and North Africa (MENA) region has the highest global prevalence of T2DM, estimated at approximately 16%, while estimates for Iran range between 8.1% and 14.2%, as reported by various researchers.^3-5^ Among the numerous modifiable risk factors for T2DM, dietary habits play a critical role in both prevention and management.^6-9^

 The Dietary Approaches to Stop Hypertension (DASH) diet, which includes vegetables, fruits, whole grains, lean proteins, legumes, nuts, and low-fat dairy products, is rich in essential nutrients such as fiber, potassium, magnesium, and antioxidants.^10-12^ Originally developed to reduce hypertension, the DASH diet has since been linked to improvements in cardio-metabolic health and inflammatory biomarkers.^13-15^ This diet has gained significant attention for its role in preventing and managing insulin resistance and T2DM.^16,17^

 While numerous studies have examined the association between the DASH diet and T2DM risk and glycemic indices, the findings remain inconsistent.^18-20^ In both observational studies and randomized clinical trials, higher adherence to the DASH diet was associated with improvements in glycemic control, serum insulin levels, and insulin resistance^21-25^; however, these improvements were not reported in other randomized controlled trials.^26,27^ Moreover, two dose-response meta-analyses reported a significant inverse association between risk of T2DM and insulin resistance,^18,19^ but another systematic review and meta-analysis conducted on randomized controlled trials found no significant effect for the DASH diet on glycemic indices.^20^ In addition to these contradictions in the findings of studies on the association between the DASH diet and T2DM, the association of different indices of the DASH diet with T2DM risk remains largely unknown.

 Several DASH diet indices have been developed for epidemiological and population-based research to evaluate adherence to this healthy dietary pattern. Although these indices are designed to evaluate compliance with DASH dietary principles, they vary in their scoring methods, categorization of food groups, and the weighting of specific components.^28-32^ The five DASH diet indices are Folsom, Dixon, Mellen, Fung, and Günther. The Günther index is based solely on food items, while the Mellon index exclusively considers nutrient intake. Other indices utilize a mix of food and nutrient parameters, such as sodium, saturated fat, and total fat intake.^28-32^

 Previous systematic reviews and meta-analyses have indicated that higher adherence to the DASH diet, as assessed by the Fung and Günther indices, was associated with an 18% to 22% reduction in the risk of T2DM.^6,33-35^ However, there is limited comparative research examining the relationship between the various DASH indices and chronic disease risk. Current studies suggest that differences in scoring systems can result in variations in the strength and significance of their associations with health outcomes.^36-38^ For example, Miller et al found that higher adherence to the Mellen and Fung indices was associated with a decreased risk of colon cancer in women, while no such association was found for the Dixon and Günther indices.^37^ Similarly, Heidari et al reported that the Mellen and Günther indices were associated with breast cancer risk, whereas the Dixon and Fung indices showed no such association.^36^

 Although the DASH diet is well-recognized for its benefits to cardiometabolic health, there has been no research investigating the association between the five major indices for T2DM risk in Middle Eastern populations, where dietary habits (e.g. higher refined grain intake) might influence these associations. This study aimed to investigate the relationship between different DASH diet indices and the risk of T2DM within the Tehran Lipid and Glucose Study (TLGS), a large-scale, population-based prospective cohort study. By evaluating different DASH scoring systems, this research aims to identify which index is associated with T2DM risk in the Tehrani population.

## Materials and Methods

###  Study Design and Population

**Figure 1:**
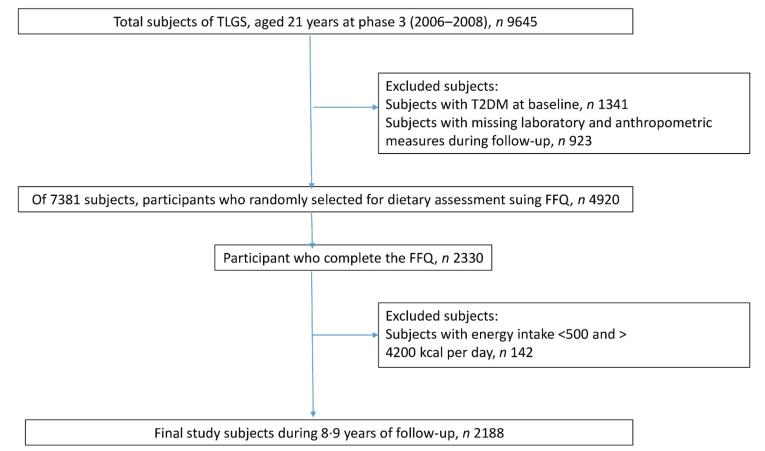
Outline of study participants’ selection

 This prospective cohort study was conducted as part of the TLGS, a longitudinal, population-based investigation in District 13 of Tehran involving 15,005 individuals aged three years and older. Previous research has extensively detailed the TLGS methodology.^39^ The study commenced in 1999, with data collection repeated every three years. Phase 3 (2005–2008) was designated as the baseline, during which participants were followed through Phase 6 (2016–2018). The inclusion criteria for this research were: (1) individuals aged 21 years or older, (2) free of diabetes at baseline, and (3) possessing complete anthropometric and laboratory data, as well as a Food Frequency Questionnaire (FFQ). Individuals with extreme energy intake ( < 500 kcal/day or > 4,200 kcal/day) were excluded from the study. Ultimately, 2,188 participants were included in the final analysis, with an average follow-up period of 8.9 years (Figure 1).

 This study adhered to ethical standards and received approval from the Institutional Review Board of the Research Institute for Endocrine Sciences at Shahid Beheshti University of Medical Sciences in Tehran, Iran. All participants provided informed consent before their inclusion in the study.

###  Dietary Assessment and DASH Diet Indexes

 Dietary intake was evaluated through face-to-face interviews conducted by trained dietitians using a validated semi-quantitative FFQ.^40-42^ Participants reported their consumption frequency of various food items over the past year, with portion sizes standardized and converted into grams. Nutrient intake, including both macronutrients and micronutrients, was calculated using the Iranian Food Composition Table.^43^

###  Folsom’s DASH Diet Index 

 The Folsom DASH Diet Index, developed by Folsom et al in 2007, assesses adherence to the DASH low-sodium dietary recommendations.^29^ Cutoff values for food groups and nutrient intakes were established based on a daily intake of 2,000 calories. In this DASH Diet Index, each item is assigned a score of 1, 0.5, or 0, reflecting the level of compliance assessed. The index emphasizes increased consumption of total grains, whole grains, vegetables, fruits, dairy products, nuts, seeds, and legumes while recommending reduced intakes of sweets, meat, poultry, fish, sodium, saturated fatty acids (SFAs), and total fat. The final score is calculated by summing the 11 components, with possible values ranging from 0 to 11 points.

###  Dixon’s DASH Diet Index

 The DASH index, developed by Dixon et al in 2007, evaluates adherence to the DASH Eating Plan as outlined in the 2005 Dietary Guidelines for Americans (Table 1).^28^ This index comprises eight food groups and one nutrient component. Participants earn one point for meeting the minimum recommended servings for specific food groups and receive zero points for not meeting these recommendations. The cutoff values for dietary intake are based on sex-specific energy needs, set at 2,000 kcal/day for men and 1,600 kcal/day for women. The final DASH index score is the sum of all nine components, resulting in a minimum score of 0 and a maximum score of 9 points.

**Table 1:** Components of Each DASH Diet Index

**Individual Component **	**Folsom Index**		**Dixon Index**		**Mellon Index **		**Fung Index**	**Günther Index**	
Total fruit	1: ≥ 4 0.5: 2‒3 0: < 2	Serving/d	≥ 4	Servings/d			Fifth quintile	≥ 4	Servings/d
Total vegetables	1: ≥ 4 0.5: 2‒3 0: < 2	Serving/d	Male ≥ 4	Servings/d				≥ 4	Servings/d
			Female ≥ 3						
Vegetables without potatoes							Fifth quintile		
Total grains	1: ≥ 7 0.5: 5‒6 0: < 5	Serving/d						≥ 6	Servings/d
Whole grains	1: ≥ 2 0.5:1 0: < 1	Serving/d	Male ≥ 4.7	Servings/d			Fifth quintile	≥ 50	% of total grain
			Female ≥ 4						
Total dairy	1: ≥ 2 0.5: 1 0: < 1	Serving/d	≥ 2	Servings/d				≥ 2	Servings/d
Low fat dairy							Fifth quintile	≥ 75	% of total dairy
Nuts, seeds, legumes	1: ≥ 4 0.5: 2‒3 0:L < 5	Serving/w	Male ≥ 4	Servings/d			Fifth quintile	≥ 4	Servings/w
			Female ≥ 3						
Meat and equivalents			< 170	gr/d					
Red meat and processed meat							Fifth quintile		
Meat, fish, poultry, egg	1: ≤ 2 0.5: 3 0: ≥ 4	Serving/d						≤ 2	Servings/d
Sweets	1: ≤ 5 0.5: 6‒7 0: ≥ 8	Serving/w						≤ 5	Servings/w
Sugar sweetened beverages							Fifth quintile		
Oils								≤ 3	Servings/d
Added sugar			≤ 3	% of total daily kcal					
Alcohol			Male ≤ 2	drink/d					
			Female ≤ 1						
Protein					1: ≥ 18 0.5: 16.5‒18 0: < 16.5	% energy intakes /d			
Fiber					1: 14.8 0.5: 9.5‒14.5 0: < 9.5	mg/1000 kcal			
Magnesium					1: 0.5: 158‒238 0:	mg/1000 kcal			
Potassium					1: 0.5: 1534‒2238 0	mg/1000 kcal			
Calcium					1: 0.5: 402‒590 0:	mg/1000 kcal			
Total fat	1: ≤ 30 0.5: 31‒32 0: ≥ 33	% energy intakes /d			1: 0.5: 27‒32 0:	% energy intakes /d			
SFA	1: ≤ 10 % 0.5: 11‒12% 0: ≥ 13 %	% energy intakes /d	≤ 5	% of total daily kcal	1: 0.5: 6‒11 0:	% energy intakes /d			
Cholesterol					1: 0.5: 71.4‒107.1 0:	mg/1000 kcal			
Sodium	1: ≤ 1500 0.5: 1501‒2400 0: ≥ 2401	mg/ d			1: 0.5: 1143‒1268 0:	mg/1000 kcal	Fifth quintile		
Total score	0-11		0–9		0-9		8‒40	0‒80	

###  Mellon’s DASH Diet Index 

 Mellen et al developed a DASH diet index in 2008 based on nutrient intakes,^32^ utilizing data from two clinical trials.^44,45^ This index evaluates adherence to the DASH diet by assessing nine key nutrients expected to be either higher (protein, fiber, magnesium, calcium, and potassium) or lower (total fat, SFA, sodium, and cholesterol) in a DASH-compliant diet (Table 1). The scoring system is based on absolute nutrient targets for a 2,100-calorie diet, applicable to both men and women. Individuals who meet the recommended goal for a given nutrient receive 1 point, those who meet an intermediate goal (defined as the midpoint between the DASH diet target and the nutrient content of the DASH control diet) receive 0.5 points, and those who fail to meet either target receive 0 points. The final DASH index score is calculated by summing the nine nutrient components, resulting in a score that ranges from a minimum of 0 to a maximum of 9 points.

###  Fung’s DASH Diet Index 

 The DASH diet index, developed by Fung et al in 2008, comprises eight components: seven food groups and one nutrient, based on the dietary recommendations outlined by the National Heart, Lung, and Blood Institute (Table 1).^30^ The scoring system employs quintile rankings to evaluate adherence to the DASH diet. Participants receive scores ranging from 1 (lowest quintile) to 5 (highest quintile) for food groups emphasized in the DASH diet. Conversely, for components that should be limited, the scoring is reversed; individuals in the highest quintile receive 1 point, while those in the lowest quintile receive 5 points. Men and women are classified into quintiles separately. The final DASH index score is calculated by summing the individual component scores, resulting in a possible range from 8 to 40 points.

###  Günther’s DASH Diet Index 

 Günther et al developed a food-based DASH diet index in 2009, consisting of 10 components to assess adherence to the DASH Eating Plan outlined in the 2005 Dietary Guidelines for Americans (Table 1).^31^ The target intakes for each component are based on recommendations for four different energy intake levels (1,600, 2,000, 2,300, and 3,100 kcal/day), taking into account age, sex, and physical activity levels as specified in the Dietary Reference Intakes. The scoring system assigns six components to a 10-point scale, while four components are scored on a 5-point scale. The total DASH index score is calculated by summing the individual component scores, with a possible range from 0 to 80 points.

###  Clinical and Laboratory Measurements

 Demographic data (including age, education level, physical activity, smoking status, marital status, medication use, and family history of T2DM) were collected using a standardized questionnaire. Body weight was measured with a digital scale, while height was recorded using a tape measure. Body mass index (BMI) was calculated as weight (kg) divided by height squared (m^2^). Waist circumference was measured at the level of the umbilicus. Physical activity was assessed using the Modifiable Activity Questionnaire, with results expressed as metabolic equivalent (MET) minutes per week.^46^ After a 15-minute rest period, systolic and diastolic blood pressure were measured twice using a mercury sphygmomanometer, and the average of the two readings was used for analysis. Fasting blood samples were collected following a 12–14-hour fast to assess fasting plasma glucose (FPG), lipid profiles, and other biomarkers. Plasma glucose and triglycerides were analyzed using an enzymatic colorimetric method, while high-density lipoprotein (HDL) cholesterol was measured after the precipitation of apolipoprotein B-containing lipoproteins. Participants who were not taking glucose-lowering medications also underwent an oral glucose tolerance test. The intra-assay and inter-assay coefficients of variation were less than 2.2% for FPG, 1.9% for triglycerides, and 2.9% for HDL cholesterol.

###  Definition of Terms

 T2DM was classified according to the criteria established by the American Diabetes Association (ADA). This classification includes FPG levels of ≥ 126 mg/dL, a 2-hour post-challenge plasma glucose (2-h-PCPG) level of ≥ 200 mg/dL, or a documented history of glucose-lowering medication use.^47^

###  Statistical Analysis

 The sample size was determined based on the hazard ratio (HR) from a previous meta-analysis, which indicated that high adherence to the DASH diet was linked to an 18% reduction in the risk of developing T2DM (HR = 0.82).^18^ The calculation included a 95% confidence level (α = 0.05), a study power of 80% (β = 0.20), and a baseline T2DM incidence of 10.8% within the Iranian adult population.^48^ The minimum sample size required to detect a hazard ratio of 0.8 was 1,614 participants. In the current study, the final analysis included 2,188 participants, which provided adequate power to evaluate the effect of adherence to the DASH diet on T2DM risk. Phase III (2005–2008) was designated as the baseline, and participants were followed through Phase VI (2016–2018). The event date for T2DM cases was described as the middle time between the follow-up visit date at which T2DM was detected for the first time; follow-up time was drawn from the difference between the calculated mid-time date and the date at which the subjects entered the study. For the censored subjects, the survival time was the time from entry into the study to either the loss to follow-up point, death from any cause, or the end of the study without having T2DM.

 The normality assumption of each variable was checked using the Kolmogorov-Smirnov test and a histogram chart. Data are presented as mean (standard deviation) and median (interquartile range (IQR)) for continuous variables, while categorical variables are expressed as percentages. The baseline characteristics and dietary intakes were analyzed using one-way analysis of variance (ANOVA) for normally distributed variables, the Kruskal-Wallis test for skewed variables, and chi-square tests for categorical variables.

 Cox proportional hazards models were employed to estimate HRs and 95% confidence intervals (CIs) for the risk of T2DM based on the median DASH diet. The proportionality-hazard assumption was evaluated using the Schoenfeld residual test using STATA (version 12; STATA Inc., TX, USA). All proportionality assumptions were generally satisfied (all *P* values were more than 0.05). Statistical models were developed as follows: Model 1 was crude. Model 2 adjusted for age, sex, total energy intake, physical activity, cholesterol consumption, and diabetes risk score. Model 3 included an additional adjustment for BMI. The diabetes risk score was determined based on five variables: systolic blood pressure, family history of diabetes, waist-to-height ratio, triglyceride-to-HDL ratio, and FPG level.^49^ Potential confounding variables were identified through a literature review and confirmed by statistical evidence. A univariate analysis was performed to evaluate these potential confounders, and variables with a *P-*value for entry (P_E_) of less than 0.2 were included as confounders in the multivariable model. This approach is also recommended by Maldonado and Greenland, using a liberal criterion of P < 0.2 for covariate inclusion.^50,51^ All statistical tests were considered statistically significant for *P* values < 0.05, and analyses were conducted using SPSS version 18.

## Results

 Of the 2,188 participants, 989 (45.2%) were men. The mean (SD) age and BMI of the population were 40.6 (13.3) years and 27.0 (4.7) kg/m^2^, respectively. During a median follow-up of 8.9 years, 234 new cases of T2DM were recorded. The censoring proportion is 1,954 participants (89.3%). The median score (IQR) of adherences to DASH diet was 6.5 (3-9.5) for Folsom, 3 (1‒7) for Dixon, 3.5 (1‒7.5) for Mellon, 24 (11‒37) for Fung, and 50 (10‒80) for Günther.

 The baseline characteristics of participants based on DASH score indices are presented in Table 2. Participants with higher adherence to the Folsom DASH index were older, had a higher BMI and diabetes risk score. Those with higher adherence to the DASH Dixon index included a larger proportion of females, had higher BMI, were less educated, smoked less, and were more likely to be married. Regarding the Mellen index, participants with higher adherence were older, more educated, had higher BMI, and smoked less. For the Fung index, individuals with higher adherence were older, had a higher BMI and diabetes risk score. The Günther index was characterized by a higher proportion of females, older individuals, lower education levels, and reduced smoking rates.

**Table 2:** Baseline Characteristics of Participants Across Medians of DASH Indices

	**Folsom DASH Index**		**Dixon DASH Index**		**Mellon DASH Index**		**Fung DASH Index**		**Günther DASH Index**	
	**<Median**	**≥Median**	**<Median**	**≥Median**	**<Median**	**≥Median**	**<Median**	**≥Median**	**<Median**	**≥Median**
Female	714 (54%)	485 (56%)	609 (44%)	590 (73%)	679 (56%)	520 (53%)	660 (55%)	539 (55%)	727 (47.5%)	472 (72%)
*P* value	0.219		< 0.0001		0.091		0.487		< 0.0001	
Age (year)	39.6 ± 13.2	42.1 ± 13.3	38.9 ± 13.5	43.4 ± 12.6	38.4 ± 12.4	43.3 ± 13.9	38.8 ± 12.8	42.7 ± 13.7	39.9 ± 13.7	42.2 ± 12.4
*P* value	< 0.0001		< 0.0001		< 0.0001		< 0.0001		< 0.0001	
Academic degrees	356 (27%)	238 (27%)	405 (29%)	189 (24%)	300 (25%)	294 (30%)	321 (27%)	273 (28%)	434 (28%)	160 (24%)
*P* value	0.496		< 0.0001		< 0.0001		0.070		0.057	
Marital status, married	1019 (77%)	678 (78%)	1052 (76%)	645 (80%)	923 (76%)	774 (79%)	927 (77%)	770 (78%)	1197 (78%)	500 (76%)
*P* value	0.327		0.010		0.061		0.209		0.136	
Smoker	172 (13%)	100 (11.5%)	204 (15%)	68 (8.5%)	169 (14%)	103 (10.5%)	164 (14%)	108 (11%)	213 (14%)	59 (9%)
*P* value	0.537		< 0.0001		0.041		0.161		< 0.0001	
Physical activity (MET min-week)	14.9 (2.9, 39.5)	17.4 (4.5, 42.0)	13.9 (2.5, 41.2)	19.0 (6.9, 41.7)	15.6 (3.0, 41.7)	16.9 (4.0, 40.6)	15.5 (2.9, 40.6)	16.7 (4.5, 41.7)	13.9 (2.8, 39.7)	19.9 (6.9, 42.6)
*P* value	0.091		0.001		0.518		0.255		< 0.0001	
BMI	26.8 ± 4.6	27.4 ± 4.8	26.5 ± 4.6	27.9 ± 4.9	26.7 ± 4.6	27.4 ± 4.8	26.6 ± 4.5	27.6 ± 4.9	26.6 ± 4.6	28.0 ± 4.8
*P* value	0.005		< 0.0001		< 0.0001		< 0.0001		< 0.0001	
Diabetes risk score	16.0 (11.0, 23.0)	16.0 (11.0, 26.0)	16.0 (11.0, 23.0)	16.0 (11.0, 26.0)	16.0 (11.0, 23.0)	16.0 (11.0, 26.0)	16.0 (11.0, 23.0)	16.0 (11.0, 26.0)	16.0 (11.0, 23.0)	16.0 (11.0, 26.0)
*P* value	0.004		0.303		< 0.0001		0.003		0.757	

Data are presented as n (%) for categorical variables, mean ± SD for normally distributed variables, and median (IQR) for skewed variables. *P* value reported using one-way analysis of variance (ANOVA) for normally distributed variables, Kruskal-Wallis test for skewed variables, and chi-square tests for categorical data.

Table 3 presents a comprehensive overview of baseline dietary intakes of nutrients and food groups based on the median cut-off for each DASH index. Participants with higher DASH index scores had higher consumption of energy, protein, carbohydrate, fiber, fat, SFA, fiber and sodium. In terms of MUFA, and PUFA, all individuals with higher scores on the DASH indices had higher consumption except for the Fung index. Participants with higher DASH index scores consumed higher amounts of whole grains, fruits, vegetables, nuts, seeds, and legumes across all indices. There was an increase in dairy product consumption among participants with higher scores in all DASH indices, except for the Günther index, where dairy product consumption decreased. Consumption of red and processed meats was increased among those with higher Folsom, Dixon, and Günther DASH scores. Conversely, individuals with higher adherence to the DASH diet, as indicated by the Mellen, Fung, and Günther indices, consumed fewer sweets. Regarding oil consumption, participants with higher adherence to the DASH diet according to the Folsom and Dixon indices reported higher daily oil intake, while those adhering to the Fung and Günther indices consumed less.

**Table 3:** Baseline Dietary Intakes of Participants Across Medians of DASH Indexes

	**Folsom DASH Index**		**Dixon DASH Index**		**Mellon DASH Index**		**Fung DASH Index**		**Günther DASH Index**	
	**<Median**	**≥Median**	**<Median**	**≥Median**	**<Median**	**≥Median**	**<Median**	**≥Median**	**<Median**	**≥Median**
Nutrients										
Total energy (kcal/d)	2017.9 ± 665.9	2592.9 ± 655.1	2111.6 ± 699.8	2477.8 ± 692.2	2286.7 ± 710.4	2195.6 ± 726.3	2154.8 ± 736.8	2358.0 ± 680.1	2165.3 ± 717.9	2433.7 ± 685.8
*P *value	< 0.0001		< 0.0001		< 0.0001		< 0.0001		< 0.0001	
Carbohydrate (g/d)	287.8 ± 103.9	379.8 ± 106.7	300.8 ± 107.3	364.9 ± 114.6	311.7 ± 105.4	339.9 ± 122.6	307.2 ± 114.5	345.3 ± 110.5	308.9 ± 110.8	360.2 ± 114.3
*P *value	< 0.0001		< 0.0001		< 0.0001		< 0.0001		< 0.0001	
Protein (g/d)	68.1 ± 25.8	89.3 ± 25.1	71.9 ± 26.9	84.5 ± 26.8	74.5 ± 26.2	79.0 ± 28.9	71.4 ± 26.6	82.8 ± 27.3	73.7 ± 27.5	83.3 ± 26.5
*P *value	< 0.0001		< 0.0001		< 0.0001		< 0.0001		< 0.0001	
Fat (g/d)	70.8 ± 29.8	88.7 ± 30.4	73.9 ± 30.8	84.6 ± 30.9	88.1 ± 32.1	65.3 ± 24.9	76.1 ± 32.5	80.1 ± 29.6	76.2 ± 31.6	83.2 ± 26.5
*P *value	< 0.0001		< 0.0001		< 0.0001		< 0.0001		< 0.0001	
PUFA (g/d)	14.9 ± 7.6	18.2 ± 8.1	15.5 ± 7.6	17.6 ± 8.3	18.6 ± 8.4	13.3 ± 6.3	16.3 ± 8.4	16.2 ± 7.4	15.8 ± 7.7	17.1 ± 8.3
*P *value	< 0.0001		< 0.0001		< 0.0001		0.744		< 0.0001	
MUFA (g/d)	24.7 ± 11.0	30.3 ± 11.1	25.7 ± 11.2	29.1 ± 11.5	30.8 ± 11.7	22.2 ± 8.9	26.7 ± 11.9	27.3 ± 10.7	26.4 ± 11.3	28.1 ± 11.4
*P *value	< 0.0001		< 0.0001		0.002		0.153		< 0.0001	
SFA (g/d)	21.2 (16.3, 28.9)	27.5 (21.7, 35.6)	22.6 (17.2, 30.6)	26.0 (20.0, 34.0)	27.6 (20.6, 36.3)	20.7 (15.9, 26.0)	22.8 (16.9, 31.0)	24.9 (19.4, 32.6)	23.2 (17.6, 31.5)	25.1 (19.3, 32.6)
*P *value	< 0.0001		< 0.0001		< 0.0001		< 0.0001		0.001	
Fiber (g/d)	27.7 (19.9, 39.9)	40.5 (31.3, 52.9)	28.9 (20.4, 42.4)	38.9 (29.7, 50.5)	30.6 (22.0, 42.6)	36.1 (25.3, 49.8)	29.6 (20.6, 44.0)	35.6 (27.0, 47.3)	29.8 (20.9, 43.2)	38.6 (29.5, 51.2)
*P *value	< 0.0001		< 0.0001		< 0.0001		< 0.0001		< 0.0001	
Cholesterol (mg/d)	177.4 (124.7, 245.7)	225.1 (170.2, 296.2)	189.1 (131.8, 258.5)	205.7 (155.6, 283.3)	219.4 (162.5, 302.5)	169.4 (124.6, 227.1)	191.6 (132.2, 267.1)	198.5 (150.3, 270.6)	191.7 (135.9, 265.5)	201.4 (154.3, 272.8)
*P *value	0.065		< 0.0001		0.011		< 0.0001		0.032	
Sodium (mg/d)	3205.3 (2347.1, 4831.5)	3763.6 (2783.6, 5559.1)	3319.6 (2357.5, 4888.8)	3700.1 (2739.7, 5541.8)	3557.4 (2583.6, 5315.9)	3316.9 (2388.6, 5076.6)	3747.2 (2533.4, 6624.9)	3168.6 (2423.7, 4334.0)	3363.1 (2390.0, 4992.0)	3635.9 (2710.9, 5506.6)
*P *value	< 0.0001		< 0.0001		0.004		< 0.0001		< 0.0001	
Food groups										
Whole grain (serving/d)	1.2 (0.6, 2.5)	2.9 (1.7, 4.5)	1.5 (0.7, 3.0)	2.5 (1.1, 4.8)	1.5 (0.7, 3.1)	2.1 (1.1, 4.1)	1.3 (0.6, 2.8)	2.5 (1.3, 4.3)	1.6 (0.8, 3.3)	2.1 (1.0, 4.3)
*P *value	< 0.0001		< 0.0001		< 0.0001		< 0.0001		< 0.0001	
Total grain (serving /d)	8.6 ± 5.1	10.3 ± 4.9	9.1 ± 5.2	9.5 ± 5.2	8.7 ± 4.0	9.9 ± 6.2	9.4 ± 5.4	9.1 ± 4.8	9.2 ± 5.3	9.5 ± 4.9
*P *value	< 0.0001		0.096		0.227		0.260		0.237	
Vegetables (serving /d)	2.9 ± 1.8	4.9 ± 2.6	3.2 ± 2.0	4.6 ± 2.7	3.5 ± 2	4.0 ± 2.7	2.9 ± 2.0	4.7 ± 2.5	3.4 ± 2.1	4.5 ± 2.8
*P *value	< 0.0001		< 0.0001		< 0.0001		< 0.0001		< 0.0001	
Fruits (serving /d)	2.1 ± 1.9	4.1 ± 2.5	1.9 ± 1.3	4.5 ± 2.9	2.5 ± 1.9	3.4 ± 2.7	2.0 ± 1.6	3.9 ± 2.7	2.3 ± 1.9	4.2 ± 2.8
*P *value	< 0.0001		< 0.0001		< 0.0001		< 0.0001		< 0.0001	
Dairy products (serving /d)	1.9 ± 1.3	2.7 ± 1.3	2.0 ± 1.3	2.5 ± 1.4	2.2 ± 1.3	2.3 ± 1.4	1.9 ± 1.2	2.6 ± 1.4	3.1 ± 1.3	2.4 ± 1.3
*P *value	< 0.0001		< 0.0001		< 0.0001		< 0.0001		0.034	
Red and Processed meat (serving/d)	0.3 (0.2, 0.6)	0.4 (0.2, 0.7)	0.4 (0.2, 0.6)	0.4 (0.2, 0.7)	0.4 (0.3, 0.8)	0.3 (0.2, 0.5)	0.5 (0.3, 0.8)	0.3 (0.2, 0.5)	0.4 (0.2, 0.6)	0.4 (0.3, 0.7)
*P *value	< 0.0001		0.021		< 0.0001		< 0.0001		0.015	
Meat, Fish, Poultry, egg (serving/d)	1.2 ± 0.9	1.3 ± 0.8	1.2 ± 0.9	1.3 ± 0.9	1.4 ± 0.9	1.2 ± 0.9	1.3 ± 0.9	1.2 ± 0.9	1.2 ± 0.9	1.3 ± 0.8
*P *value	0.001		0.121		0.190		0.025		< 0.0001	
Nuts, seeds, Legumes (serving/d)	0.2 (0.1, 0.3)	0.4 (0.2, 0.7)	0.2 (0.1, 0.4)	0.4 (0.2, 0.7)	0.3 (0.1, 0.4)	0.3 (0.1, 0.5)	0.2 (0.1, 0.3)	0.3 (0.2, 0.6)	0.2 (0.1, 0.4)	0.4 (0.2, 0.7)
*P *value	< 0.0001		< 0.0001		0.525		< 0.0001		< 0.0001	
Sweets (serving/d)	4.3 (2.5, 7.8)	4.5 (2.4, 9.3)	4.4 (2.5, 8.5)	4.3 (2.5, 7.9)	4.6 (2.7, 8.7)	3.9 (2.2, 7.5)	5.5 (2.9, 10.6)	3.4 (1.2, 6.0)	4.5 (2.5, 8.6)	4.1 (2.3, 7.7)
*P *value	0.276		0.629		< 0.0001		< 0.0001		0.023	
Oil (serving /d)	1.9 (1.1, 2.8)	2.1 (1.3, 3.2)	2.0 (1.1, 2.9)	2.1 (1.2, 3.1)	2.5 (1.8, 3.7)	1.3 (0.8, 1.3)	2.1 (1.2, 3.0)	1.9 (1.1, 2.9)	2.1 (1.1, 3.1)	1.9 (1.1, 2.7)
*P *value	< 0.0001		0.105		< 0.0001		0.019		0.020	

Data are presented as n (%) for categorical variables, mean ± SD for normally distributed variables, and median (IQR) for skewed variables. *P* value reported using one-way analysis of variance (ANOVA) for normally distributed variables, Kruskal-Wallis test for skewed variables, and chi-square tests for categorical data.

Table 4 displays the HRs for the risk of T2DM across the upper and lower medians of DASH indices. In Model 1, there was no association between T2DM and participants in the upper versus lower medians across all DASH indices. Adjusting for age, sex, total energy intake, physical activity, cholesterol intake, and diabetes risk score in Model 2 did not change these associations. However, after additional adjustment for BMI in Model 3, an inverse association was observed between the Dixon index (HR = 0.71; 95% CI: 0.52‒0.98) and the Günther index (HR = 0.69; 95% CI: 0.50‒0.96) with the risk of T2DM.

**Table 4:** Multivariable Adjusted Hazard Ratio (95% CI) for T2DM Across the Median Cut-off in Different DASH Indexes

	**Folsom DASH index**		**Dixon DASH Index**		**Mellon DASH Index**		**Fung DASH Index**		**Günther DASH Index**	
	**<Median**	**≥Median**	**<Median**	**≥Median**	**<Median**	**≥Median**	**<Median**	**≥Median**	**<Median**	**≥Median**
Model 1	1.00	1.09 (0.84, 1.41)	1.00	1.02 (0.78, 1.33)	1.00	1.45 (1.12, 1.88)	1.00	1.20 (0.92, 1.55)	1.00	0.95 (0.72, 1.27)
Model 2	1.00	0.97 (0.72, 1.30)	1.00	0.81 (0.59, 1.11)	1.00	1.18 (0.91, 1.54)	1.00	1.01 (0.77, 1.31)	1.00	0.77 (0.56, 1.06)
Model 3	1.00	0.90 (0.67, 1.21)	1.00	0.71 (0.52, 0.98)	1.00	1.23 (0.94, 1.60) *	1.00	0.90 (0.68, 1.18)	1.00	0.69 (0.50, 0.96)

Model 1: crude. Model 2: Model 1 + adjustment for age, sex, total energy, physical activity, cholesterol intake, and diabetic risk score. Model 3: Model 2 + adjustment for BMI at baseline. As cholesterol intake is a component of the Mellon index, it was not adjusted in calculating the multivariable adjusted hazard ratio for this index.

**Figure 2:**
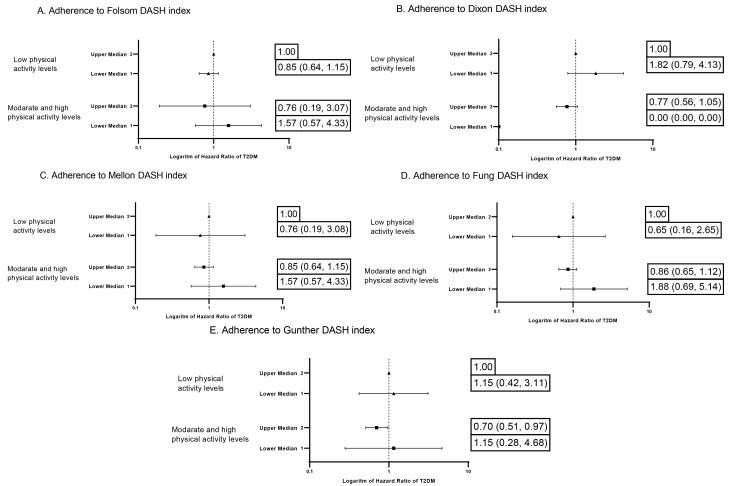
Hazard Ratios of the DASH Diet Indices and Physical Activity Levels on Riskof T2DM after Adjustment for Age, Sex, Total Energy Intake, Cholesterol Intake, Diabetes Risk Score, and BMI

 Additionally, an interaction was observed between the DASH diet scores and physical activity, BMI, and weight change. Therefore, we evaluated the modifying effects of these variables on the association between the DASH diet score and the risk of T2DM using multivariable Cox regression models. No association was found between DASH indices and the risk of T2DM across BMI status (normal, overweight, and obese) and weight changes ( ≥ 3% weight gain, ± 3% weight stability, ≥ 3% weight loss). Figure 2 illustrates the risk of T2DM and the DASH diet indices, stratified by physical activity levels. Günther index adherence reduced the risk of T2DM only in low-activity individuals (HR = 0.70, 95% CI: 0.51–0.97). In other indices, we could not find any association between the risk of T2DM and physical activity levels.

## Discussion

 In this prospective cohort study, adherence to the DASH diet, when assessed using the Dixon and Günther indices, was associated with a reduced risk of T2DM. However, adherence to the Folsom, Mellon, and Fung indices did not associate with incident T2DM. These findings emphasize the potential role of diet quality in the prevention of T2DM, while also highlighting the variability in results depending on the specific DASH index employed.

 The DASH dietary pattern has been emphasized for its benefits to cardiometabolic health. Consistent with our findings, several systematic reviews and meta-analyses have demonstrated that adherence to the DASH diet is associated with a reduced risk of T2DM and other cardiometabolic diseases.^6,33-35^ Despite the findings of a systematic review that suggested no significant effect for the DASH diet on improvement in FBG and insulin resistance,^19,20^ cumulative evidence [44–49] suggests that the DASH diet prevents T2DM.^18^ In pooled meta-analyses on observational studies, high adherence to the DASH diet was significantly associated with a marginal inverse association in cohort studies (RR. 83; 95% CI: 0.76‒0.91, *P* < 0.001).^18^ However, high heterogeneity was found in the aforementioned study (*P* < 0.0001; I2 = 89.1%).^18^ Adherence to the DASH diet in prospective studies reduces the risk of T2DM from 25% to 69% during 5 to 20 years of follow-up. This risk reduction among the Asian population, in the Singapore Chinese Health Study, was 29% (HR = 0.71, 0.65‒0.79).^52^ In the US population, the Health Professionals Follow-Up Study showed a 25% reduction (HR = 0.75; 95% CI: 0.65–0.85),^53^ while the Insulin Resistance Atherosclerosis Study in whites reported a 69% reduction (HR = 0.31; 95% CI: 0.13–0.75).^54^ However, this association was not observed among blacks or Hispanics^54^ and in Taiwan.^55^ The greatest risk reduction was found using the Günther index of the DASH diet^54^; risk reduction using the Fung index was 30%.^52,54^ In the current study, we found that, consistent with previous studies, the Dixon DASH index and Günther index reduced the risk of T2DM by 30% (HR = 0.71 for the Dixon index and 0.69 for the Günther index), representing a clinically meaningful risk reduction of developing T2DM at a population level.^56^ From a practical standpoint, our findings suggest that promoting adherence to the DASH diet, particularly as defined by the Günther or Dixon indices, could be a valuable component of public health strategies for T2DM prevention in the Tehrani adult population.

 The observed differences among the five DASH indices can be attributed to their distinct methodological approaches. Each index varies in its emphasis on specific food groups, nutrient intake, and scoring systems, which influences its ability to capture dietary adherence and predict health outcomes. The Dixon and Günther indices, which exhibited significant inverse associations with T2DM risk due to their known anti-inflammatory and insulin-sensitizing effects, have unique scoring criteria that may better align with dietary patterns in this population, whereas nutrient-focused indices (e.g. Mellen) may overlook synergistic food interactions.^36-38^ The Günther index employs a broader scoring range (0 to 80), potentially providing a more nuanced assessment of dietary adherence.^31^ In contrast, other indices, such as the Folsom (0 to 11), Mellen (0 to 9), and Fung (8 to 40) indices, utilize different scoring scales and weightings, which may account for their lack of association with T2DM in this study.^29,30,32^ The Günther index emphasizes food group intake, while other indices either use a combination of food groups and nutrients or emphasize nutrient intake entirely.^28-30,32^ The most important difference between the Dixon and Günther indices and other indices is their scoring system. The Günther and Dixon indices provide different cutoff points for calorie intake based on age, gender, and physical activity level,^28,31^ while other indices either use fixed cutoff points^29,30^ or emphasize intake density.^32^

 Population characteristics played a crucial role in shaping these findings. Participants who demonstrated greater adherence to the Dixon and Günther indices were more likely to be female, married, and less educated. They had healthier lifestyles, were non-smokers, and had lower diabetic risk scores. These demographic differences suggest that adherence to specific dietary patterns may vary based on sociodemographic factors, potentially impacting disease risk.^57,58^ Furthermore, the overall dietary patterns observed in this Iranian cohort differed from those in Western populations, where most DASH indices were initially developed and validated. For example, while higher adherence to the DASH diet is generally associated with a higher intake of whole grains, fruits, vegetables, nuts, and legumes, certain variations were noted. Participants with higher Günther and Dixon scores consumed more red and processed meat, whereas those with higher Folsom and Dixon scores had a higher intake of oils. Such variations in food consumption may contribute to the differing predictive abilities of the indices.

 An important finding was the influence of physical activity on the relationship between the DASH diet and the risk of T2DM. Among individuals with lower levels of physical activity, higher adherence to the Günther index was associated with a reduced risk of T2DM. However, no significant interactions were observed for other indices based on age, BMI, or weight change. This suggests that dietary quality, as measured by the Günther index, may be particularly beneficial for physically inactive individuals, potentially offsetting the adverse effects of a sedentary lifestyle. The study lacks sufficient power to assess interactions in moderate and high physical activity groups due to the limited cases.

 Our study has several strengths. To our knowledge, this is the first study to investigate the association between five established DASH diet indices and T2DM. The prospective cohort design, relatively large sample size, and extended follow-up period enhance the reliability of our findings. Additionally, we employed a validated food frequency questionnaire to assess dietary intake and had access to comprehensive data on a range of potential confounders. However, there are several limitations to consider. The reliance on self-reported dietary intake through the FFQ is subject to measurement error and recall bias, which may lead to misclassification of participants’ adherence to the DASH diet. However, using the validation FFQ as well as alternative methods provides a more accurate evaluation of long-term dietary habits. Although we adjusted for a wide array of confounders, residual confounding due to unmeasured or imprecisely measured factors (e.g. genetic predisposition, environmental exposures, or other lifestyle variables) cannot be ruled out. Furthermore, our study population consisted of Tehrani adults; thus, the generalizability of our findings to other populations with different dietary patterns and genetic backgrounds may be limited.

## Conclusion

 In conclusion, our study identified a significant association between adherence to the DASH diet and the incidence of T2DM among Tehrani adults; however, this association was not consistent across all DASH indices. Several factors may account for these discrepancies, including the distinct purposes for which each index was developed, variations in scoring systems, and differences in food consumption patterns within the Tehrani population. From a practical standpoint, our findings suggest that promoting adherence to the DASH diet, particularly as defined by the Günther or Dixon indices, could be a valuable component of public health strategies for T2DM prevention in the Tehrani adult population. The Günther index may be the most suitable measure, given its methodological characteristics and the specific attributes of the study population, to enhance the understanding of the DASH diet’s impact on T2DM prevention.
